# Crash Risk Prediction Modeling Based on the Traffic Conflict Technique and a Microscopic Simulation for Freeway Interchange Merging Areas

**DOI:** 10.3390/ijerph13111157

**Published:** 2016-11-19

**Authors:** Shen Li, Qiaojun Xiang, Yongfeng Ma, Xin Gu, Han Li

**Affiliations:** School of Transportation, Southeast University, Si Pai Lou #2, Nanjing 210096, China; happylishen@126.com (S.L.); xqj@seu.edu.cn (Q.X.); guxin0307@126.com (X.G.); lhfrostring@126.com (H.L.)

**Keywords:** traffic safety, traffic conflict, traffic simulation, interchange merging area

## Abstract

This paper evaluates the traffic safety of freeway interchange merging areas based on the traffic conflict technique. The hourly composite risk indexes (HCRI) was defined. By the use of unmanned aerial vehicle (UAV) photography and video processing techniques, the conflict type and severity was judged. Time to collision (TTC) was determined with the traffic conflict evaluation index. Then, the TTC severity threshold was determined. Quantizing the weight of the conflict by direct losses of different severities of freeway traffic accidents, the calculated weight of the HCRI can be obtained. Calibration of the relevant parameters of the micro-simulation simulator VISSIM is conducted by the travel time according to the field data. Variables are placed into orthogonal tables at different levels. On the basis of this table, the trajectory file of every traffic condition is simulated, and then submitted into a surrogate safety assessment model (SSAM), identifying the number of hourly traffic conflicts in the merging area, a statistic of HCRI. Moreover, the multivariate linear regression model was presented and validated to study the relationship between HCRI and the influencing variables. A comparison between the HCRI model and the hourly conflicts ratio (HCR), without weight, shows that the HCRI model fitting degree was obviously higher than the HCR. This will be a reference to design and implement operational planners.

## 1. Introduction

Ramp junctions are points at which on- and off-ramps join the freeway. The junction formed at this point is an area of turbulence due to the concentration of merging or diverging vehicles. A well-designed on-ramp should allow vehicles to merge on the main route efficiently and safely; thus, various contributing factors should be considered, including acceleration lane speed, acceleration lane length, traffic volume, etc. [1]. Many previous studies have focused on on-ramp traffic control and capacity improvement [2,3,4,5].

The most frequently observed types of crashes at merging areas are side collisions and rear-end collisions, where vehicle performance, driver expectations, driver performance, and freeway geometry interact. Numerous studies have been conducted on the safety evaluation and improvement of merging areas with the help of accident data or surrogate safety measures through simulations or field observations. Sadia has presented an interchange complexity model (ICM) that was based on estimations of aggregated drivers’ workload at interchanges, to estimate the complexity of interchange design alternatives and the implications for road safety to minimize driver errors and, thus, reduce crash occurrences [6]. Torbic used the spreadsheet-based interchange safety analysis tool (ISAT) for the safety performance functions to identify substantive gaps in the current state of knowledge that limit the ability of ISAT to provide all of the capabilities desired by potential users [7]. Lu compared the prediction performance between the simple and full safety performance function (SPF) models for estimating the data collected on urban four-lane freeway interchange influence areas in Florida, supported by the use of the flow-only SPF model adopted in *Safety Analyst* [8,9]. Fan had used the surrogate safety assessment model (SSAM) and VISSIM for safety assessment at freeway merge areas. The simulated conflicts generated by the VISSIM simulation models and identified by the SSAM approach those measured in the field using the traditional traffic conflict technique. A two-stage procedure was proposed to calibrate and validate the VISSIM simulation models, and data analysis results have shown that there was a reasonable consistency between the simulated and the observed conflicts [10,11,12].

In most conventional studies, traffic accident data were widely used to analyze the safety condition of traffic facilities [13,14]. In China, due to the low availability of traffic accident data, the feasibility of an accident-based evaluation method is greatly restricted. It is urgent to develop a surrogate safety measure to assess the traffic facilities and traffic running conditions. As a non-accident traffic safety measure, the traffic conflict technique has been widely applied, especially at urban intersections [15,16,17,18,19,20,21,22,23,24,25]. However, the validity of the traffic conflict technique on the expressway, in the field, has not been fully substantiated, especially with respect to the application to the interchange segment. It has long been difficult to collect traffic conflict data from freeway interchanges due to the lack of observation points. Recently, the rapid development of unmanned aerial vehicle (UAV) photography and video-based trajectory tracking techniques has provided a reliable guarantee for the acquisition of traffic conflict data from interchanges.

This paper starts from simple merging forms on a freeway interchange and selects a directional ramp as the object of study. It is concluded that the outer lane of the mainline is obviously disturbed by the on-ramp, and discussed emphatically with respect to traffic safety of the merging area by the influence of the outer lane and the acceleration lane. The objective of this study is to evaluate the traffic safety of freeway interchange merging areas based on the traffic conflict technique. The hourly composite risk indexes (HCRI) was defined. Moreover, the multivariate linear regression model was presented and validated to study the relationship between HCRI and the influencing variables.

## 2. Methodology

### 2.1. Time to Collision (TTC) Calculation

In a freeway interchange area, when two or more vehicles run near each other within a certain period of time, one side must take an avoidance measure to change the vehicle’ moving status; otherwise, a conflict will occur. An interchange area is an uninterrupted traffic facility on which the angle between the vehicle traveling directions is less than 90°.

Two typical types of traffic conflicts were defined: (1) lane-change conflict, the angle between two vehicles belongs to (15°, 85°); and (2) rear-end conflict, the angle between two vehicles belongs to (0°, 15°).

#### 2.1.1. The Rear-End Conflict

**Figure 1:**
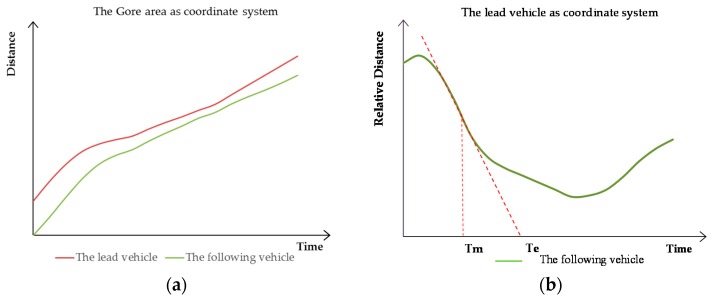
The time-distance variation chart of rear-end conflict. (**a**) The state of the two vehicles’ absolute travel distance with time (the gore area as the coordinate system); (**b**) The state of the following vehicle’s relative travel distance with time (the lead vehicle as the coordinate system).

The rear-end conflict location is in the same lane. The lead vehicle’s speed has slowed suddenly, and the two vehicles are fast approaching; if one does not take measures, a collision will likely occur. Figure 1 depicts a plot of the time-distance change graph. In Figure 1a, the distance between the vehicles decreases gradually with time, but alternative measures are taken, and the distance increased gradually. In Figure 1b, using the lead vehicle as the coordinate system, the following vehicle’s relative travel distance decreased quickly with time before taking measures.

During the rear-end conflict process, the largest slope of the relative distance, with respect to time, is recorded as $T_{M}$. At this time, if measures have not been taken, the following vehicle will hit the lead one at this relative speed. The virtual collision moment is recorded as $T_{e}$.

The rear-end conflict TTC calculation method is shown in Equation (1):

(1) $$TTC_{R}=|T_{e}-T_{M}|$$

where

$TTC_{R}$—Rear-end conflict value, s;
$T_{M}$—The largest slope of Relative Distance occurred time, s;
$T_{e}$—The virtual collision moment, s.

#### 2.1.2. The Lane-Change Conflict

**Figure 2:**
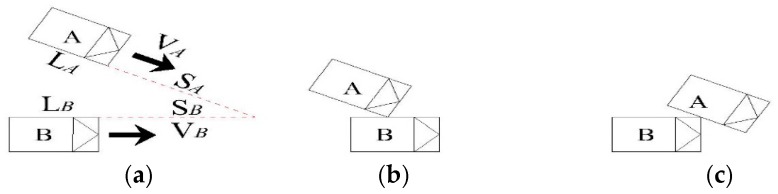
The lane-change conflict process diagram. (**a**) Avoidance behavior generation time; (**b**) the potential collision point while vehicle B is running faster; and (**c**) the potential collision point while vehicle A is running faster.

The lane-change conflict location is not in the same lane. An alternative vehicle has changed its direction for overtaking, entering the off-ramp or mainline. When there is another following vehicle along the original lane, there is the trajectory cross-point between the two vehicles. When the two vehicles are fast approaching, if one does not take measures, a collision will likely occur. The lane-change conflict process is shown in Figure 2.

The avoidance behavior generation is denoted as $T_{0}$, while the alternative vehicle speed has changed significantly. At this time, measuring the speed, the vehicle whose direction changed is recorded as $V_{A}$, and the vehicle whose direction stayed the same is recorded as $V_{B}$. Then the distance of the avoidance behavior generation site of the vehicle whose direction changed to the trajectory cross-point is recorded as $S_{A}$, and the vehicle whose direction stayed the same is recorded as $S_{B}$. The vehicles’ body lengths, recorded as $L_{A}$ and $L_{B}$, are measured, respectively. The travel time of the vehicle whose direction changed from the *T*_0_ site to the trajectory cross-point is denoted as $T_{A}=S_{A}/V_{A}$. The vehicle whose direction stayed the same is denoted as $T_{B}=S_{B}/V_{B}$. The lane-change conflict TTC calculation method is shown in Equations (2) and (3):

When $T_{A}\geq T_{B}$,

(2) $$\{\begin{array}{c} \mathrm{If}\;T_{A}\leq T_{B}+L_{B}/V_{B},\\ TTC_{L}=T_{A}\\ \mathrm{Otherwise},\;\mathrm{no}\;\mathrm{conflict}. \end{array}$$

When $T_{A}\leq T_{B}$,

(3) $$\{\begin{array}{c} \mathrm{If}\;T_{B}\leq T_{A}+L_{A}/V_{A},\\ TTC_{L}=T_{B}\;\\ \mathrm{Otherwise},\;\mathrm{no}\;\mathrm{conflict}. \end{array}$$

where

$TTC_{L}$—Lane-change conflict value, s;
$T_{0}$—Avoidance behavior generation time, s;
$L_{A}$—Vehicle A length, m;
$L_{B}$—Vehicle B length, m;
$V_{A}$—Vehicle A speed at $T_{0}$, m/s;
$V_{B}$—Vehicle B speed at $T_{0}$, m/s;
$S_{A}$—Travel distance of vehicle A from avoidance behavior generation site to trajectory cross-point, m;
$S_{B}$—Travel distance of vehicle B from avoidance behavior generation site to trajectory cross-point, m;
$T_{A}$—Travel time of vehicle A from avoidance behavior generation site to trajectory cross-point, s;
$T_{B}$—Travel time of vehicle B from avoidance behavior generation site to trajectory cross-point, s.

#### 2.1.3. TTC Threshold Calculation

##### Severity Classification

According to the conflict angle, determine the type of conflict; according to the observers’ common judgment, the severity was divided into general and serious.

##### TTC Value Calculation

Using the method described in Section 2.1.1, calculate the rear-end conflict TTC value, or the lane-change in Section 2.1.2.

##### TTC Threshold Calculation

For example, all of the serious lane-change conflict TTC values were divided into equal intervals, and statistics were accumulated, taking the 85th percentile value as the upper limit threshold of the class.

### 2.2. HCRI Calculation

In [26] there is morphological data concerning the accidents occurring on the expressways in Chongqing during a period of five years. In detail, according to the conflict angle, the crashes occurring in (15°, 85°) can be considered as lane-change conflict accidents, while the conflict accidents occurring in (0°, 15°) can be considered as rear-end conflict accidents. Values are assigned to the degree of passenger injury in accordance with the direct loss ceiling specified in the standard traffic accident classification table [27], 200 for the direct loss caused by a minor accident, 5000 for the direct loss by a general accident, and 10,000 for the direct loss by a major accident (the mean loss of general and major accidents is equal to 7500). The direct loss caused by accidents is estimated by type, as shown in Table 1.

Therefore, a weight of 0.54 is assigned to rear-end conflicts, while a weight of 0.46 is assigned to lane-change conflicts. Generally speaking, a wide-angle lane-change conflict contributes more to fatalities than a rear-end conflict since there is not a sufficient vehicle body to absorb the conflict energy. However, since a lane-change conflict usually occurs with both vehicles involved giving way to each other, and the conflict speed is rarely as high as a fast rear-end conflict speed, the accident severity is relatively low. Thus, this weight is logical and considered fit for this study.

TTC is the time to collision, a measurement closely related to accident severity. The closer the TTC is to 0, the more dangerous it will be, and when TTC = 0, a crash will have occurred. Therefore, the weight of the different conflict risk degrees could be determined according to the reciprocal proportion of the average TTC at various severity levels. The average TTC value and the weight of different severity levels are shown in Table 2.

The HCRI is calculated in Equation (4):

(4) $$\mathrm{HCRI}=\left(L_{S}\times 0.65+L_{G}\times 0.35\right)\times 0.46+\left(R_{S}\times 0.62+R_{G}\times 0.38\right)\times 0.54$$

where

HCRI—Hourly conflict risk indexes;
$L_{S}$—The number of serious lane-change conflicts;
$L_{G}$—The number of general lane-change conflicts;
$R_{S}$—The number of serious rear-end conflicts;
$R_{G}$—The number of general rear-end conflicts.

The numbers of various conflicts are multiplied by their respective weight and multiplied by their type weight.

If the number of conflicts is used directly, the hourly total volume conflict ratio is recorded as the hourly traffic collision rate (HCR), and the total volume is composed of the outer volume and the on-ramp volume.

### 2.3. Model Prototype

According to the literature [28,29,30], a number of the following factors, including the outer lane capacity in the merging area, ramp capacity, acceleration lane length, percent of heavy vehicles on the ramps, percent of heavy vehicles in the outer lane, and the designed ramp speed, are adopted in accordance with the previous studies to analyze the traffic safety in the merging area.

Multivariate linear regression is a theoretical method to study the relationship between a dependent variable and multiple independent variables. The model prototype is shown in Equation (5):

(5) $$HCRI=\sum \alpha_{i}X_{i}+\beta$$

where

HCRI—Hourly conflict risk indexes;
$\alpha_{i}$—Independent variable coefficient;
$X_{i}$—Some independent variable;
$X_{1}$—Ramp traffic volume, veh/h;
$X_{2}$—Outer lane traffic volume, veh/h;
$X_{3}$—Acceleration lane length, m;
$X_{4}$—Ramp percent heavy vehicles, %;
$X_{5}$—Outer lane percent heavy vehicles, %;
$X_{6}$—Ramp design speed, km/h.
β—Constant term.

## 3. Data Collection

### 3.1. Field Data

**Figure 3:**
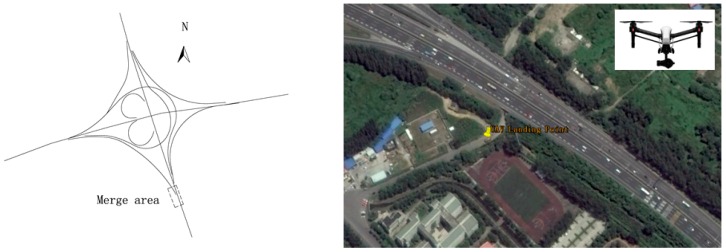
Maqun interchange investigation site.

The traffic survey site was selected at the Maqun interchange in northeast Nanjing. It is a semi-cloverleaf interchange type, with a typical diverging, merging, and weaving area. The investigation site is shown in Figure 3. UAV photography was allowed to shoot video in the necessary surroundings, including the wind force below grade 4, sunny weather, good light, and no electromagnetic interference. The UAV could hover for 10 min at maximum effectiveness. The survey received a total of 25 videos.

#### 3.1.1. Observer Training

Traffic conflict observers were trained based on certain knowledge. Firstly, 12 traffic conflict observers were requested to watch traffic accident videos and grasp the forming mechanism of traffic accidents. Then, they practiced making judgments until all of them obtained the same result for the same conflict video. Finally, they worked together to judge the types and severity of the traffic conflict videos.

#### 3.1.2. Data Processing

##### The Rear-End Conflict

Tracker is a free video analysis and modeling tool built on the Open Source Physics (OSP) Java framework [31]. Features include object tracking with position, velocity and acceleration overlays, and graphs, special effect filters, multiple reference frames, calibration points, line profiles for analysis of spectra and interference patterns, and dynamic particle models.

**Figure 4:**
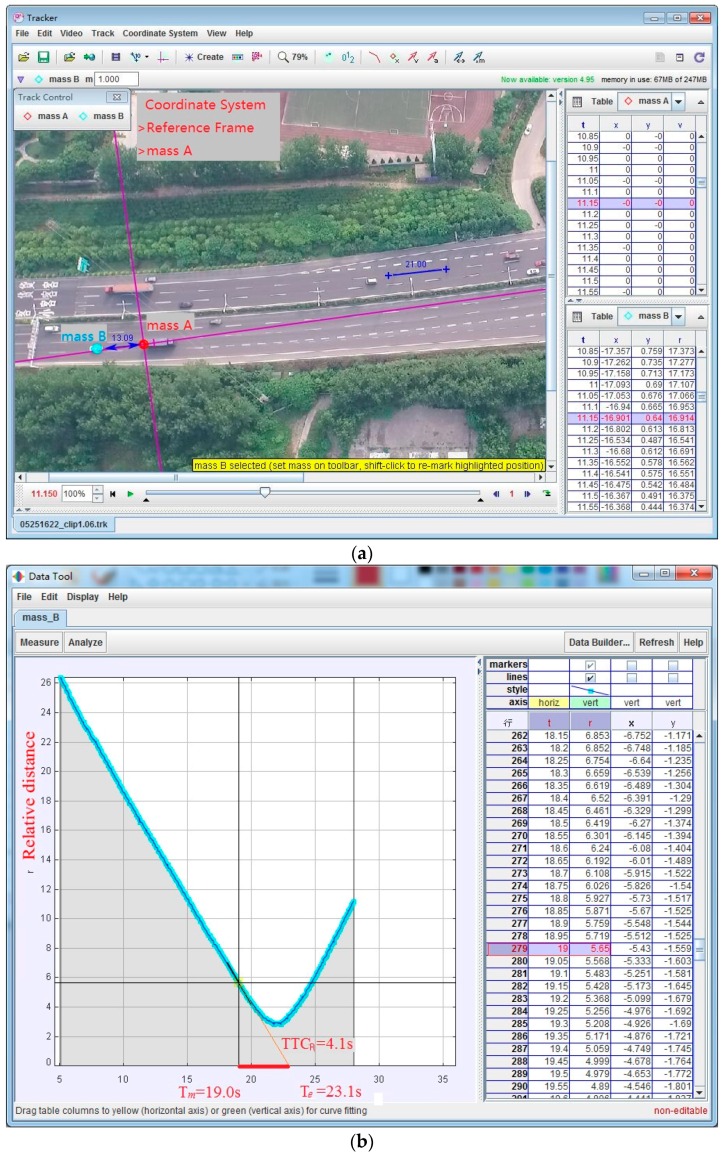
The rear-end conflict TTC calculation in Tracker. (**a**) The conflict vehicles’ speed in Tracker (the lead vehicle as the coordinate system); and (**b**) the rear-end conflict TTC calculation demonstration in Tracker.

In Tracker, we set the gore area as the coordinate reference system, correcting the size dimensions by highway traffic signs and markings. Taking the lead vehicle’s track, and then taking the lead (mass A) as the coordinates to record the relative track of the following vehicle, the processing of the relative distance is shown in Figure 4a. The rear-end conflict TTC calculation is shown in Figure 4b, e.g., $TTC_{R}=|T_{e}-T_{M}|$ = 23.1 − 19.0 = 4.1 s.

##### The Lane-Change Conflict

**Figure 5:**
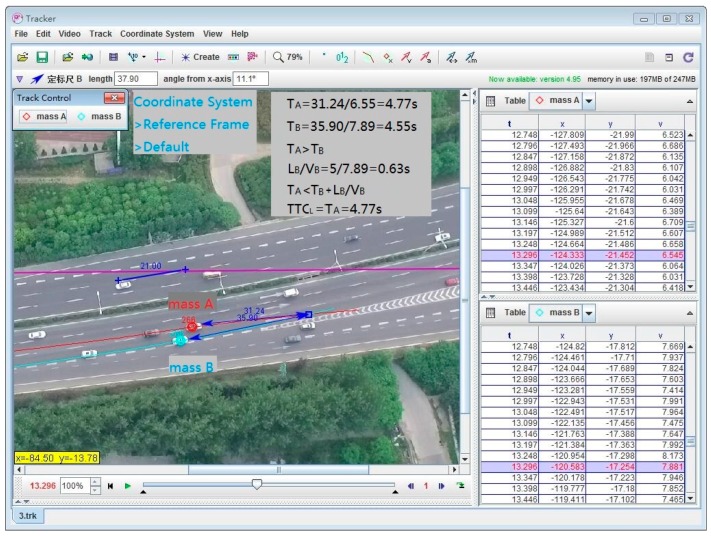
The lane-change conflict TTC calculation in Tracker.

We set the gore area as the coordinate reference system, and adjusted the size dimensions by highway traffic signs and markings. *V_A_* and *V_B_*, respectively, are the avoidance behavior generation times, and $S_{A}$ and $S_{B}$, respectively, are the travel distances from the avoidance behavior generation site to the trajectory cross-point. The lane-change conflict *TTC_L_* calculation is shown in Figure 5, e.g., $TTC_{L}=T_{A}$ = 4.77 s.

##### TTC Threshold Calculation

After processing some fault data, the remaining data were divided into equal intervals. The 85th percentile value was adopted as the upper limit for the TTC value. The thresholds of serious and general rear-end and lane-change conflicts were calculated, respectively. The conflict thresholds are shown in Table 3.

### 3.2. Simulation Data

#### 3.2.1. Calibration and Validation

Twenty-five datasets of the Maqun interchange were observed. Twenty datasets were used to calibrate the simulation parameters. The other five datasets were used to validate the simulation.

Microscopic simulation model validation is a discrete selection optimization problem, and the most common genetic algorithm. Its advantage lies mainly in the use of space coding parameters to perform the operation, rather than the parameter itself, and it can quickly obtain the overall best solution, but also avoids falling into the partial optimal solution [32].

Then driving behavior parameters are calibrated by the genetic algorithm, and the sum of the squares of the errors of travel time is used as the fitness function. The calibration process can be divided into three parts.

##### Part 1

In VISSIM, build the road model, including the input lane, vehicle, path selection, vehicle composition, expected speed distribution, and so on.

##### Part 2

**Figure 6:**
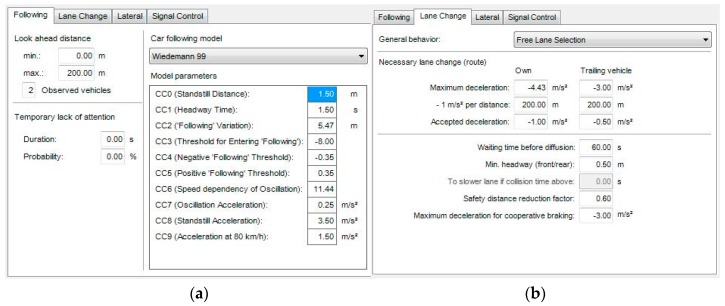
The simulation parameters calibration: (**a**) car following model; and (**b**) lane-change model.

Use C# to realize the genetic algorithm to calibrate the driving behavior parameters automatically, to seek the fitness function to be minimized, and to calibrate the output parameters. The results of the calibration of driving behavior parameters in the confluence area are shown in Figure 6.

##### Part 3

Operating the vehicle trajectory file of the simulation in SSAM to observe the statistical number of conflicts. When the calculated mean absolute percentage error (MAPE) is less than or equal to 15%, it meets the requirements. Otherwise, return to Part 3 to adjust.

It was found that the relative error of travel time between the simulation model and the actual time observed is 3.141%, and the relative error of the conflict number is 12.032%, from Table 4. Therefore, the simulation data error is in the required range, and the simulation data can reflect the confluence of the actual situation.

#### 3.2.2. Simulation Design

For the analysis of the field data, the mean and standard deviations are shown in Table 5.

##### The Range of Variables

We determine the range of values for each variable based on the survey values and the extremes of the specification. The ramp traffic volume ranged from 600 to 1000 veh/h; the outer lane traffic volume ranged from 1000 to 1500 veh/h; the acceleration lane length ranged from 150 to 300 m; the percent of heavy vehicles on ramps ranged from 2% to 10%; the percent of heavy vehicles on the outer lane ranged from 5% to 40%; and the designed ramp speed ranged from 40 to 80 km/h. The variables are evenly divided into six levels, which can reflect the role of the variable, as shown in Table 6.

##### Design of Orthogonal Test Table

There are six variables in the model, each of which has six levels. If all of them are completed, the test requires 6^6^ = 46,656 times. The orthogonal test can simplify the number of tests, and is a widely used scientific method. The experiment selected L_108_ (6^11^), which only needed to be simulated 108 times, and can represent all of the cases.

##### Independent Variable Correlation

The correlation between the two variables is shown in Table 7. The correlations between the six variables are poor, and the respective characteristics can be expressed separately.

##### Data Analysis

The HCRI and HCR of 108 sets of simulation data are calculated, as shown in Table 8.

## 4. Model

### 4.1. Establishment of the Model

Multivariate linear regression models of HCR and HCRI are built from 108 sets and their dependent variables, as shown in Table 9.

The R-squared value of the HCRI model was larger than the HCR model, which indicates that the HCRI model outperforms the HCR model. As a traditional conflict evaluation index, HCR has been widely used on urban roads. HCRI can be used as a supplement for the methodology of traffic conflict research.

The non-significant variables were removed, and the regression model was built again, as shown in Table 10.

The regression model formula is shown in Equation (6):

(6) $$HCRI=0.119366\;\times X_{1}+0.381029\;\times \;X_{3}-104.1185$$

where

HCRI*—*Hourly conflict risk indexes;
$X_{1}$—Ramp traffic volume, veh/h;
$X_{3}$—Acceleration lane length, m.

When the ramp traffic volume increases, the interference increases and the risk of a traffic conflict increases. When the acceleration lane length increases, the driver’s lane-change choice increases, and the traffic conflicts also increase. This also shows that the effect of the acceleration lane is significantly greater than that of the ramp flow.

### 4.2. Model Validation

**Figure 7:**
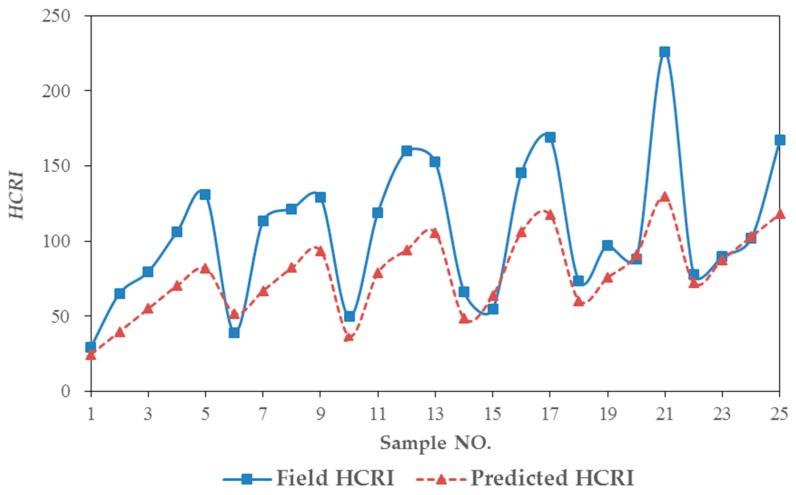
Validation and actual comparison chart.

Twenty-five datasets of the Luoxi interchange were used to calibrate the regression model. The observed HCRI were recorded as Field HCRI. The predicted value of HCRI was recorded as Predicted HCRI. The numerical analysis between Field HCRI and Predicted HCRI is shown in Table 11 and Figure 7.

The root mean square error is 38.27, the mean absolute percentage error is 25.91%, the accuracy of the model prediction is credible, and the safety status of the merging area could be predicted according to the on-ramp volume and the acceleration lane length.

In Figure 7, the numerical difference is quite obvious. The reason may be that the multivariate linear model was not appropriate. As more models are established in the future, or the variety of models increases, better results may be represented.

## 5. Conclusions

A traffic conflict model with a new index is developed in this study. The major significance of this study is summarized as follows: Firstly, the time to collision (TTC) value was calculated based on the data extracted from the UAV photography and Tracker. The TTC measurement accuracy was significantly improved. Secondly, thanks to the high quality of the data sets, the present study can offer comprehensive coverage of various variables with refined scales, adding to the understanding of traffic conflict modeling in merging areas. Finally, the proposed HCRI traffic conflict models are developed with the simulation datasets, which are commonly available on merging areas around the country. As a result, a similar method can be applied to hundreds of merging areas in the interchange.

Detailed data sets from field data and simulation data, including the outer lane capacity in the merging area, ramp capacity, acceleration lane length, percent of heavy vehicles on ramps, percent of heavy vehicles on the outer lane, and designed ramp speed, are adopted in the study. A number of critical factors about ramp capacity and acceleration lane length are found significant to HCRI. Some important findings are summarized in the following statements:

(1) The threshold of serious and general lane-change conflicts lies between 0–2.3 s and 2.3–4.2 s, respectively; the threshold of serious and general rear-end conflicts lies between 0–2.8 s and 2.8–4.7 s, respectively.
(2) The field data was used to calibrate and verify the simulation model. The traffic conflict MAPE is 12.032%, which meets the requirements. The fitting degree of the HCRI model is 0.620. The HCRI model can be used as an effective complement to the method of the traffic conflict technique.
(3) The MAPE of the verified model was 25.91%. More models on the HCRI index can be established in the future.
(4) This paper reports the explorative effort on developing a new traffic conflict model using HCRI as an evaluation index. Such a study bears a lot of potential for engineering applications and safety evaluation.

## Figures and Tables

**Table 1:** Traffic conflict type weight.

Total No.	Accident Type	Total Loss	Average Loss	Weight
92	Rear-end	820,000	8913.04	0.54
90	Lane-change	675,000	7500.00	0.46

**Table 2:** Traffic conflict severity weight.

Conflict Type	Rear-End Conflict		Lane-Change Conflict	
	Serious (S)	General (G)	Serious (S)	General (G)
TTC average value	2.4	3.9	1.8	3.3
Severity Weight	0.62	0.38	0.65	0.35

**Table 3:** Interchange traffic conflict thresholds.

Conflict Type	Rear End		Lane-Change	
	Serious Conflict	General Conflict	Serious Conflict	General Conflict
Threshold (TTC/s)	0–2.8	2.8–4.7	0–2.3	2.3–4.2

**Table 4:** Summary statistics of the observed and simulated results for the calibration dataset.

Validation Sets	Travel Time			The Number of Conflicts		
	Observed	Simulated	$MAPE_{ATT}$	Observed	Simulated	$MAPE_{CC}$
1	11.4	11.2	1.754%	72	65	9.722%
2	10.3	10.5	1.942%	94	85	9.574%
3	10.8	11	1.852%	35	41	17.143%
4	9.6	10.1	5.208%	35	40	14.286%
5	10.1	10.6	4.950%	53	58	9.434%
Average	10.44	10.68	3.141%	57.8	57.8	12.032%

Note: $MAPE_{ATT}$ denotes the mean absolute percent error of the average travel time; $MAPE_{CC}$ denotes the mean absolute percent error of the average conflicts.

**Table 5:** The means and standard deviations of the field data (Maqun interchange).

No.	X_1_ (veh/h)	X_2_ (veh/h)	X_3_ (m)	X_4_ (%)	X_5_ (%)	X_6_ (km/h)
Mean	865	1250	180	0.07	0.26	62.54
Std. deviation	155	245	45	0.02	0.13	13.25

Note: Mean denotes the average value. Std. deviation denotes standard deviation.

**Table 6:** The range of each variable.

No.	X_1_ (veh/h)	X_2_ (veh/h)	X_3_ (m)	X_4_ (%)	X_5_ (%)	X_6_ (km/h)
1	500	1000	150	0.02	0.05	40
2	600	1100	180	0.04	0.10	48
3	700	1200	210	0.06	0.20	56
4	800	1300	240	0.08	0.30	64
5	900	1400	270	0.10	0.40	72
6	1000	1500	300	0.12	0.50	80

**Table 7:** The variable correlations.

Variable	X_1_	X_2_	X_3_	X_4_	X_5_	X_6_
X_1_	1.0000					
X_2_	−0.0397	1.0000				
X_3_	0.0000	−0.0079	1.0000			
X_4_	−0.0000	0.0238	0.0000	1.0000		
X_5_	−0.0000	0.0241	−0.0000	−0.0000	1.0000	
X_6_	0.0000	−0.0397	0.0000	0.0000	0.0000	1.0000

**Table 8:** The mean and standard deviation of simulation data.

Evalution Index	Maximum	Minimum	Mean	Std. Deviation
HCRI	147.953	3.095	71.137	35.980
HCR	0.329	0.008	0.162	0.079

Note: Mean denotes the average value. Std. deviation denotes standard deviation. HCRI denotes the hourly composite risk indexes. HCR denotes the hourly traffic collision rate.

**Table 9:** Regression model parameters analysis for the HCR and HCRI models.

Variable	HCR			HCRI		
	Coef.	Std. Err.	*p* > |t|	Coef.	Std. Err.	*p* > |t|
X_1_	0.000227	0.000030	0.000	0.120002	0.012565	0.000
X_2_	−0.000026	0.000030	0.396	0.016022	0.012587	0.206
X_3_	0.000838	0.000100	0.000	0.381452	0.041851	0.000
X_4_	−0.090874	0.150695	0.548	−49.579750	62.791880	0.432
X_5_	0.060689	0.032330	0.063	23.210860	13.471470	0.088
X_6_	−0.000608	0.000377	0.110	−0.215238	0.157059	0.174
_cons	−0.137401	0.057390	0.019	−114.403800	23.913310	0.000
R-squared	0.5450			0.620		
Root MSE	0.05348			22.283		

Note: Coef. denotes coefficient; Std. Err. denotes standard error of estimate ; *p* value denotes the variable significant; t denotes terminal; _cons denotes constant term; Root MSE denotes root mean square error.

**Table 10:** The regression model after removing the non-significant variables.

HCRI	Removing X_2_, X_4_, X_6_			Removing X_2_, X_4_, X_6_, X_5_		
	Coef.	Std. Err.	*p* > |t|	Coef.	Std. Err.	*p* > |t|
X_1_	0.119367	0.012628	0.000	0.119366	0.012750	0.000
X_3_	0.381029	0.042094	0.000	0.381029	0.042501	0.000
X_5_	23.625440	13.54627	0.084			
_cons	−110.2217	14.01081	0.000	−104.1185	13.69801	0.000
R-squared	0.6156			0.6081		
MSE	22.413			22.63		

Note: Coef. denotes coefficient; Std. Err. denotes standard error of estimate; *p* value denotes the variable significant; t denotes terminal; _cons denotes constant term; Root MSE denotes root mean square error.

**Table 11:** The means and standard deviations of the field data (Luoxi interchange).

Validation Dataset	Maximum	Minimum	Mean	Std. Deviation	RMSE	MAPE
Field HCRI	225.70	29.28	106.08	46.90	38.27	25.91%
Predicted HCRI	129.56	24.66	78.25	27.11		

Note: Mean denotes the average value. Std. deviation denotes standard deviation. RMSE denotes root mean square error; and MAPE denotes mean absolute percentage error.
